# Numerical Simulations of the Motion and Deformation of Three RBCs during Poiseuille Flow through a Constricted Vessel Using IB-LBM

**DOI:** 10.1155/2018/9425375

**Published:** 2018-02-21

**Authors:** Rongyang Wang, Yikun Wei, Chuanyu Wu, Liang Sun, Wenguang Zheng

**Affiliations:** ^1^Faculty of Mechanical Engineering and Automation, Zhejiang Sci-Tech University, Hangzhou 310018, China; ^2^College of Mechanical and Electrical Engineering, Huzhou Vocational and Technical College, Huzhou 313000, China; ^3^School of Mechanical Engineering and Automation, University of Science and Technology Liaoning, Anshan 114051, China

## Abstract

The immersed boundary-lattice Boltzmann method (IB-LBM) was used to examine the motion and deformation of three elastic red blood cells (RBCs) during Poiseuille flow through constricted microchannels. The objective was to determine the effects of the degree of constriction and the Reynolds (Re) number of the flow on the physical characteristics of the RBCs. It was found that, with decreasing constriction ratio, the RBCs experienced greater forced deformation as they squeezed through the constriction area compared to at other parts of the microchannel. It was also observed that a longer time was required for the RBCs to squeeze through a narrower constriction. The RBCs subsequently regained a stable shape and gradually migrated toward the centerline of the flow beyond the constriction area. However, a sick RBC was observed to be incapable of passing through a constricted vessel with a constriction ratio ≤1/3 for Re numbers below 0.40.

## 1. Introduction

Red blood cells (RBCs) play an important role in blood flow in the human body, particularly in the transportation of oxygen from the lungs to every cell of the body. An adult RBC has a biconcave shape of diameter 6 *μ*m and thickness 2 *μ*m [1–6]. The RBC membrane is highly deformable, which enables the passage of RBCs through a blood vessel with a diameter smaller than that of the RBCs [7, 8]. The flow of RBCs through a blood vessel represents a typical fluid-structure interaction (FSI) problem, involving a complex interplay of fluid dynamics, elastic body, and a moving boundary [9]. A variety of accurate and efficient numerical methods have been proposed for the solution of a FSI problem involving a complex geometry, such as the arbitrary Lagrangian–Eulerian method [10], immersed interface method [11], immersed finite element method [12], immersed boundary method [13], and immersed boundary-lattice Boltzmann method (IB-LBM) [14–18].

Previous studies on the IB-LBM emphasized its potential advantages for the solution of FSI problems, namely, its simplicity, parallelizability, intrinsic kinetic and explicit calculations, and essential relative simplicity for handing complex, moving, and deformable geometries [14–18]. In recent years, the numerical investigation of the motion and deformation of RBCs in capillaries and arteries has received considerable attention [15, 16]. Zhang et al. [4] presented a numerical IB-LBM algorithm for investigating the microscopic hemodynamic and hemorheological behaviors of discrete RBCs in shear flows. Dadvand et al. [9] used the IB-LBM to numerically investigate the motion and deformation of healthy and sick RBCs in viscous shear flows. Shi et al. [19] proposed a two-dimensional (2D) elastic spring model of the RBC membrane based on the immersed boundary method, which was first introduced by Peskin [20] for the investigation of blood flow through heart valves. Krüger et al. [21] used a hybrid LB-IB-finite element method to simulate the tumbling and tank-treading-like motion of dense suspended RBCs in an external shear flow. The transient motion and deformation of healthy RBCs and PF-RBCs at different stages were examined in a simple 2D microchannel, with the RBCs moving along the center line of the channel [22, 23]. Sui et al. [24–26] used a combination of the IBM, a multiblock lattice Boltzmann model, and membrane mechanics to investigate the transient behaviors of elastic capsules and the deformation and aggregation of RBCs in a shear flow. Ma et al. [27] proposed an IB-LBM that considered the ultrasonic effect for the simulation of RBC aggregation and deformation in an ultrasonic field. They found that the action of the ultrasound waves on the pure plasma could induce a recirculation flow. The IB-LBM has also been used to numerically investigate the effect of the RBC deformability on the dispersion of the cells at physiological flow rates with respect to the hematocrit [28]. Further, the IB-LBM has been applied to quantitative analyses of the motion and deformation of the RBC membrane in a Poiseuille flow and its compression during passage through a stenotic microvessel, with a focus on the cell-cell interaction strength [2, 3, 29]. The flow of multiple RBCs through a microvascular bifurcation has also been simulated by the 2D IB-LBM and an RBC spring model [30, 31]. Other methods have been used for the same purpose, such as by Stamou and Buick [32] and Wang et al. [33]. Alizadeh et al. [17, 18] also used a hybrid IB-LBM to investigate the dynamics of healthy and sick RBCs during flows through a constricted vessel. The foregoing shows that the IB-LBM is effective for investigating the dynamics of RBCs in flows through constricted vessels and in relevant biomedical applications.

The present study represents further work about certain previous studies [30, 31], namely, an examination of the motion and deformation of RBCs by numerical simulation using the IB-LBM. The primary objective was a qualitative analysis of the effects of the degree of constriction in the vessel and the Re number on the physical characteristics of flowing RBCs. The RBC dynamics were extensively analyzed with respect to the degree of constriction, Re number, elastic modulus, and bending modulus. The IB-LBM was specifically used to examine the physical characteristics of three elastic RBCs. Flows through a simple straight vessel and a vessel with an annular bump were considered. The rest of this paper is organized as follows.  Section 2 briefly describes the employed governing equations and numerical method. The detailed numerical results are presented and discussed in Section 3. Finally, the conclusions drawn from the study and the scope for further study are presented in Section 4.

## 2. Governing Equations and Numerical Method

### 2.1. Governing Equations

Consider an RBC with curved boundary Γ immersed in the 2D viscous fluid domain *Ω*. The point on RBC boundary Γ is characterized by the Lagrangian parameters **X**(*s*, *t*), and the fluid domain *Ω* is represented by the Eulerian coordinates **x**. The equations governing the incompressible flow and elasticity of the RBCs in an external force field are as follows [17, 18]:

(1) ρ∂u∂t+u·∇u=−∇p+μ∇2u+fx,t

(2) ∇·u=0

(3) fx,t=∫ΓFs,t·δx−Xs,tds.

In the above equations, *ρ*, **u**, *p*, and *μ*, respectively, denote the fluid density, fluid velocity, fluid pressure, and dynamic viscosity; **f**(**x**, *t*) and **F**(*s*, *t*) are, respectively, the membrane forces acting on the RBCs at the Eulerian point **x**(*x*, *t*) and Lagrangian point **X**(*s*, *t*); and *δ*(**x** − **X**(*s*, *t*)) is a nondimensional Dirac delta function.

### 2.2. Immersed Boundary-Lattice Boltzmann Method (IB-LBM)

A popular kinetic model, namely, the discrete Boltzmann equation in the Bhatnagar–Gross–Krook (BGK) model with a single relaxation time under an external force, may be reproduced as follows [9, 18, 22, 27, 29]:

(4) fαx+eαδt,t+δt−fαr,t=−1τfαx,t−fαeqx,t+δt·Gα

(5) Gα=1−12τ·ωa·ea−ucs2+eα·ucs4eα·f,

where *f*_*α*_^eq^ is the equilibrium distribution function, *f*_*α*_ is the distribution function, *τ* is the single relaxation parameter, *δ*_*t*_ is the time interval, **e**_*α*_ is the particle velocity, and *ω*_*α*_ is a weight coefficient that is determined by the selected lattice velocity model. In the present study, a 2D lattice with nine velocity components, referred to as D2Q9, was employed. The formation of the D2Q9 lattice is illustrated in Figure 2.

The discrete velocity vectors of the 2D square lattice of D2Q9 can be expressed as

(6) eα=0,0α=0ccos⁡α−1π2,sin⁡α−1π2α=1,2,3,42ccos⁡2α−1π4,sin⁡2α−1π4α=5,6,7,8,

where *c*  (=*δ*_*x*_/*δ*_*t*_) is the lattice speed and *δ*_*x*_ is the lattice constant. *ω*_*α*_ are the weight coefficients with the following values:

(7) $$\begin{array}{c} \omega_{\alpha }=\left\{\begin{array}{cc} \frac{4}{9} & \alpha =0\\ \frac{1}{9} & \alpha =1,2,3,4\\ \frac{1}{36} & \alpha =5,6,7,8. \end{array}\right. \end{array}$$

The equilibrium distribution function *f*_*α*_^eq^ was chosen from the nine-velocity set model for 2D problems, as follows:

(8) $$\begin{array}{c} f_{a}^{eq}=\rho \omega_{a}\left[\;1+\frac{e_{a}\cdot u}{c_{s}^{2}}+\frac{{\left(e_{a}\cdot u\right)}^{2}}{2c_{s}^{4}}-\frac{{\left|u\right|}^{2}}{2c_{s}^{2}}\;\right], \end{array}$$

where $c_{s}=1/\sqrt{3}\cdot c$ is the speed of sound.

An immersed boundary treatment of a nonslip boundary condition was adopted, wherein the boundary force is spread to the lattice points and the fluid lattice velocity is interpolated to the boundary points [18].  Figure 3 illustrates a 2D part of the membrane and the surrounding fluid. The interaction between the blood and the RBCs can be considered based on the relationship between the Lagrangian and Eulerian points using the following interaction equations [8, 9]:

(9) fx,t=∫0lFs,tδx−Xs,tdsUs,t=uXs,t,t=∫Γux,tδXs,t−xdxδhx=δhx·δhy,

where **F**(*s*, *t*) is the Eulerian force of the fluid flow, **f**(*x*, *t*) is the Lagrangian force of the immersed boundary, and *l* represents the cross-sectional profile of the immersed boundary of a discrete RBC. *δ*(**x** − **X**(*s*, *t*)) can be smoothly approximated by a continuous kernel distribution *δ*(*x*), as proposed by Peskin [20]:

(10) $$\begin{array}{c} \delta \left(\;x\;\right)=\left\{\begin{array}{cc} 1-\left|x\right| & 0\leq \left|x\right|\leq \Delta x\\ 0 & \Delta x\leq \left|x\right|. \end{array}\right. \end{array}$$

The position of the RBC is updated explicitly:

(11) $$\begin{array}{c} \frac{\partial X\left(s,t\right)}{\partial t}=U\left(\;s,t\;\right). \end{array}$$

The macroscopic density is evaluated as *ρ* = ∑_*α*_*f*_*α*_, the velocity as **u** = (1/*ρ*)∑_*α*_*f*_*α*_**e**_*a*_, the pressure as *p* = *ρc*_*s*_^2^, and the viscosity as *ν* = (*τ* − 1/2)*c*_*s*_^2^ · *δ*_*t*_.

Equation (4) can be decomposed into the two following distinct parts that can be executed in succession.

Collision is

(12) $$\begin{array}{c} f_{\alpha }^{*}\left(\;x,t+\delta_{t}\;\right)=f_{\alpha }\left(\;x,t\;\right)-\frac{1}{\tau }\left(\;f_{\alpha }\left(\;x,t\;\right)-f_{\alpha }^{eq}\left(\;x,t\;\right)\;\right). \end{array}$$

Streaming is

(13) $$\begin{array}{c} f_{\alpha }\left(\;x+c_{\alpha }\cdot \delta_{t},t+\delta_{t}\;\right)=f_{\alpha }^{*}\left(\;x,t+\delta_{t}\;\right). \end{array}$$

Here, *f*_*α*_^*∗*^(**x**, *t* + *δ*_*t*_) represents the distribution function after the collision, with its execution followed by streaming of the resulting distribution *f*_*α*_^*∗*^(**x**, *t* + *δ*_*t*_) to neighboring nodes.

A Chapman–Enskog expansion can be used to obtain the equations of the density and momentum from (4). To derive the classical fluid equations ((1) and (2)), two macroscopic time scales (*t*_1_ = *εt* and *t*_2_ = *εt*) and a macroscopic length scale (*x*_1_ = *εx*) are required. An execution of the streaming operation on the left-hand side of each of the classical fluid equations ((1) and (2)) obtained by the Chapman–Enskog expansion can be used to determine the inertial terms.

### 2.3. RBC Model

A natural undeformed human RBC has a biconcave disk shape. The *x*-*y* coordinates of the RBC cross-sectional profile can be described by the following equation [15]:

(14) y¯=0.5×1−x¯21/2×c0+c1x¯2+c2x¯4,−1≤x¯≤1,

where *c*_0_ = 0.207, *c*_1_ = 2.002, and *c*_2_ = 1.122. A physical model of the cross-sectional profile of an RBC is shown in Figure 4.

### 2.4. Boundary Conditions

Three different boundary conditions were implemented in this study. A periodic boundary condition was applied to both the vessel inlet and outlet [1, 4, 18, 23, 29]; a nonslip boundary condition was applied to the solid-wall boundary of the vessel [7]; and a half-way bounce-back boundary condition was applied to the straight vessel walls.

The boundary conditions of the bottom and top walls are, respectively, expressed by the following equations:

(15) f3′xf,t+δt=f4xf,t,f5′xf,t+δt=f6xf,t,f8′xf,t+δt=f7xf,t,f4′xf,t+δt=f3xf,t,f6′xf,t+δt=f5xf,t,f7′xf,t+δt=f8xf,t.

The no-slip boundary condition on the fluid-solid interface is satisfied by making the velocity of any point on the solid surface equal to that of the adjacent fluid particle [9, 17, 25, 26].

## 3. Simulation Results and Discussions

**Figure 1:**
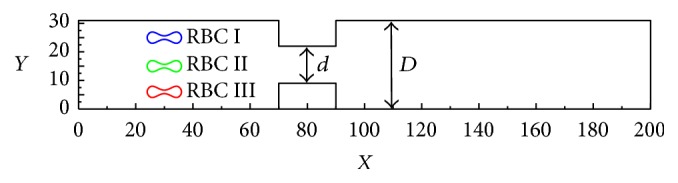
Schematic descriptions of the physical RBC models.

**Figure 2:**
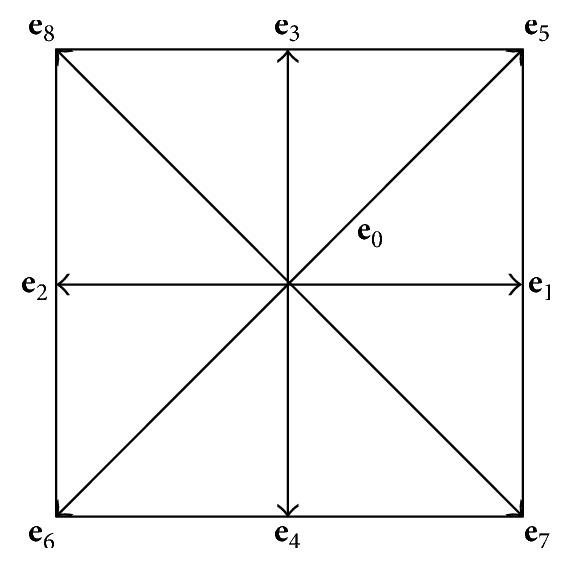
D2Q9 lattice.

**Figure 3:**
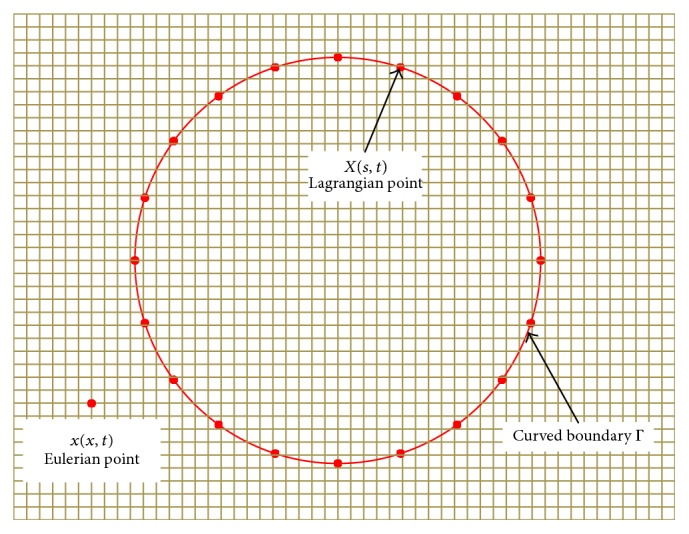
Structural boundary immersed in the 2D computational domain.

A model of a microvessel with an annular bump was constructed as shown in Figure 1. Numerical calculations were performed over 200 × 32 lattice nodes covering a physical space of 100 *μ*m × 15 *μ*m. A uniform square mesh with a nondimensional unit of *dx* = *dy* = 1 was employed. The membrane of an RBC of ≈6 *μ*m in diameter and ≈2 *μ*m in thickness was represented by 100 elastic elements. The elastic modulus *E*_*s*_ and bending modulus *E*_*b*_ were, respectively, set to 6.0 × 10^-3 ^Pa·s and 2.0 × 10^−19^ Pa·s for a healthy RBC and 6.0 × 10^-2 ^Pa·s and 2.0 × 10^−18^ Pa·s for a sick RBC, while the nondimensional unit conditions *E*_*b*_ and *E*_*s*_ were set to 0.1 and 0.001 for a healthy RBC, respectively. The nonslip boundary condition was applied to the solid-wall boundary of the channel, while the immersed RBC elastic boundary and the periodic boundary conditions were, respectively, applied to the inlet and outlet of the channel. The physical problem is governed by the nondimensional Re number defined by Re = *ρR*^2^*γ*/*μ*, where *R* is the RGBs radius and *γ* is the flow shear rate. The Re number was 0.1. To examine the motion and deformation of the three considered RBCs during flows through constricted vessels, five cases involving different degrees of constriction values (=*d/D*) were investigated. The initial positions of RBC I (upper), RBC II (middle), and RBC III (lower) were (25,30), (15,30), and (2,30), respectively (see Figure 1).

### 3.1. Motion and Deformations of “Healthy” and “Sick” RBCs in a Straight Vessel

The motions and deformations of healthy and sick RBCs in a straight vessel were compared. The elastic RBCs were placed asymmetrically in a Poiseuille shear flow near the wall of the channel.

Figure 5 shows the rotational motions of an initially spherical vesicle in Poiseuille flows, it is evident that the spherical membrane will rotate clockwise and is dragged toward the centerline of the channel, and the red bold point illustrates the rotation (Figure 5(c)). The comparison reveals a good agreement among the experimental results [34] (Figure 5(a)), numerical simulation by the FE-IBM [35] (Figure 5(b)), and the present numerical results (Figure 5(c)).

The time evolutions of the rotational motions of a healthy and sick RBC initially positioned vertically are plotted in Figure 6: (a) healthy RBC, (b) sick RBC, and (c) numerical simulation by the FE-LBM [35]. It can be observed from Figure 6(a) that a healthy RBC undergoes deformation and an unsteady tank-treading motion toward the center of the channel, attributable to the shearing effect of the Poiseuille flow.  Figure 6(b) shows profile snapshots of the sick RBC during its deformation. The sick RBC exhibits an almost steady tumbling-rotating behavior accompanied by periodical shape deformation. It rotates clockwise and preserves its shape. The above observations are well consistent with the findings of previous studies [7, 9, 26, 27, 35].

Figure 7 shows snapshots of the deformation of healthy and sick RBCs initially positioned thwart-wise ((a) and (b): current numerical simulation; (c) and (d): numerical results of [9]). The comparison reveals that the current numerical simulation results (Figures 7(a) and 7(b)) are in line with the numerical simulation by Dadvand et al. (Figures 7(c) and 7(d)) [9, 35]. Comparison of the cases of different initial states in Figures 6 and 7 suggests that the elastic modulus significantly impacts the overall mechanical characteristics of the tank-treading and tumbling-rotating motions of an RBC.

The spatial-temporal evolutions of the motions, deformations, and shapes of three healthy RBCs along the channel are shown in Figure 8. It can be observed that RBC II moves over a longer distance compared to RBCs I and III. RBC II assumes a typical arrow-like shape, whereas RBCs I and III acquire a diagonal configuration. This may be attributed to RBC II being located near the centerline of the channel, where the pressure is higher. RBCs I and III gradually migrate toward the centerline of the channel, although the latter migrates further and is more elongated.

To investigate the effect of the Re number on the variation of the barycentric coordinates, four different Re numbers, namely, 0.10, 0.15, 0.20, and 0.25, were considered. The vertical movements of the RBCs for the different Re numbers are described in Figure 9, the relationship of *t* and vertical distance is shown in Figure 9(a), and Figure 9(b) reveals the effect of Re on the barycentric coordinates. Firstly, the vertical distance increases with increasing Re number, accompanied by farther migration toward to the centerline of the channel, to reduce the flow resistance. Secondly, in a certain position, the Re has little effect on the barycentric coordinates, and the position of *X* direction has important influence on the longitudinal displacement.

### 3.2. Motion and Deformation of Three RBCs in a Constricted Vessel

The spatial-temporal evolutions of the motions and deformations of three healthy RBCs in various constricted vessels are shown in Figures 10(a)–10(f), which, respectively, correspond to cases of* d/D* = 30/30 at* t = *45 ms*, d/D* = 24/30 at* t = *47 ms,* d/D* = 20/30 at* t = *51 ms,* d/D* = 16/30 at* t* = 59.5 ms,* d/D *= 12/30 at* t* = 85 ms, and* d/D *= 10/30 at* t* = 116.5 ms. It can be seen that the RBCs in the vessel would deform and pass through the constricted part easily. Ahead of the constriction, the RBCs are swept by the fluid flow with minimal deformation. As they approach the constriction, they rotate toward the center of the flow and gradually assume an approximately horizontal orientation. The RBCs are relaxed and vibrate elastically, although the vibration rapidly decays under the viscous damping of the surrounding fluid. The RBCs subsequently regain their stable shape within a short time.

Comparison of the six constriction cases suggests that, with increasing constriction, the RBCs are forced to exhibit higher deformability than in other parts of the microchannel to squeeze through the constriction. In addition, a longer time is required for the RBCs to squeeze through a narrower constriction, attributable to the greater deformation required. It is also noteworthy that the initial position of the RBCs is not on the centerline of the channel, although they migrate toward the centerline, assuming a diagonal configuration to reduce the flow resistance.

In contrast with a healthy RBC, the elastic modulus of a sick RBC is reduced to 0.05 while the other parameters are maintained constant.  Figure 11 shows the spatial-temporal evolutions of the motions and deformations of three sick RBCs in a constricted vessel. Figures 11(b)–11(e) reveal that the sick RBCs easily pass through the constriction and no obstruction will occur during the process, and the overall characteristics of the motions are similar consistent with healthy RBCs. However, the ultimate shape of the sick RBCs significantly defers from that of the healthy RBCs in Figures 10(b)–10(e), and this is attributed to the variations of the pressure along the flow direction. It can also be seen that the sick RBCs move slower than the healthy one due to its larger elastic module. Moreover, for a low constriction ratio of* d/D* = 1/3 in Figure 11(f), only the sick RBC II emerges from the constriction zone, with the sick RBCs I and III touching the boundary of the constriction, and this touching phenomenon can produce friction, which caused the RBCs aggregating at the constriction area.

The deformations and motions of three sick RBCs in a constricted vessel with* d*/*D* = 1/3 are shown in Figure 12. It can be observed that, with increasing Re number up to 0.40, the RBCs pass through the constriction region and gradually migrate toward the centerline of the channel. This is due to the fact that, with the Re number increasing, the shear force acting on the RBCs could undergo a bigger growth, and then the RBCs will experience more deformation. People who have suffered a hypertensive disease experienced a crucial augment in blood velocity, which maybe caused fractures and lacerations. Heart disease may cause a reduction in blood velocity, under the low-speed vessels which maybe caused deoxygenation [18]. In addition, the elastic modulus, blood pressure, flow velocity, and Re number significantly impact the passage of the RBCs passing through a constricted vessel.

### 3.3. Effect of Constriction Ratio on RBC Mechanical Behavior

**Figure 4:**
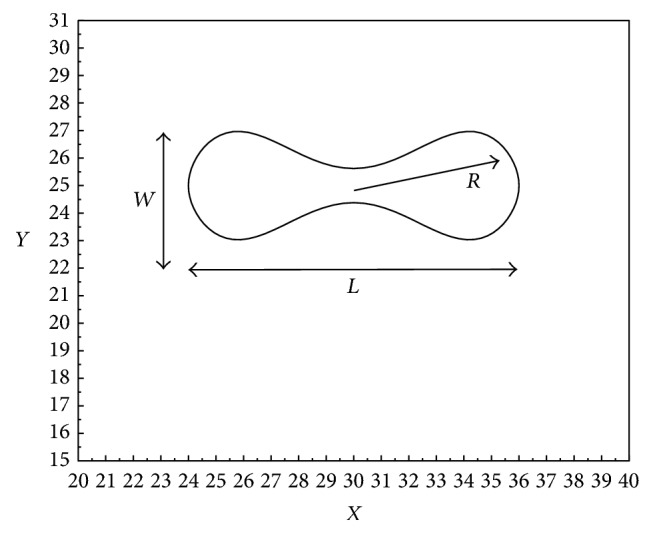
Physical model of the cross-sectional profile of an RBC of length* L*, width* W*, and radius* R*.

**Figure 5:**
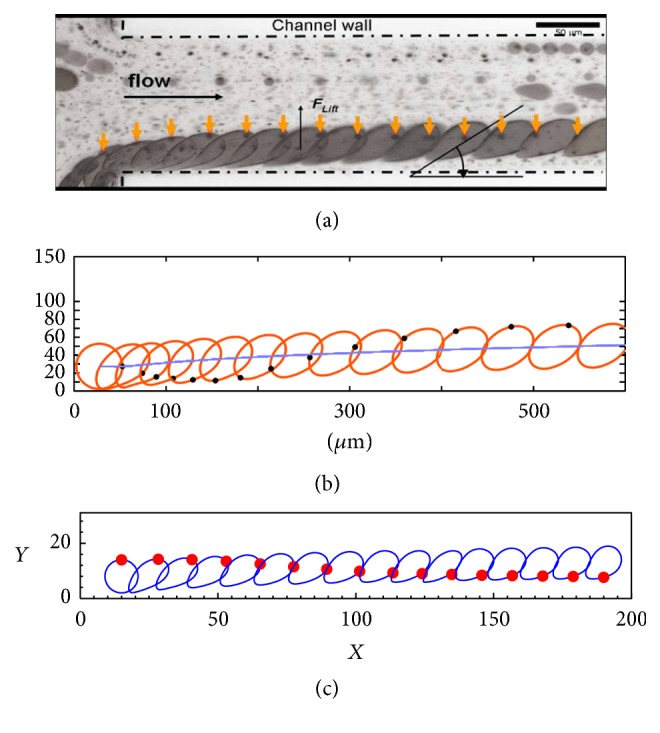
The rotational motions of an initially spherical vesicle in Poiseuille flows: (a) series of snapshots from experimental data [34], (b) numerical simulation by the FE-LBM [35], and (c) current numerical simulation.

**Figure 6:**
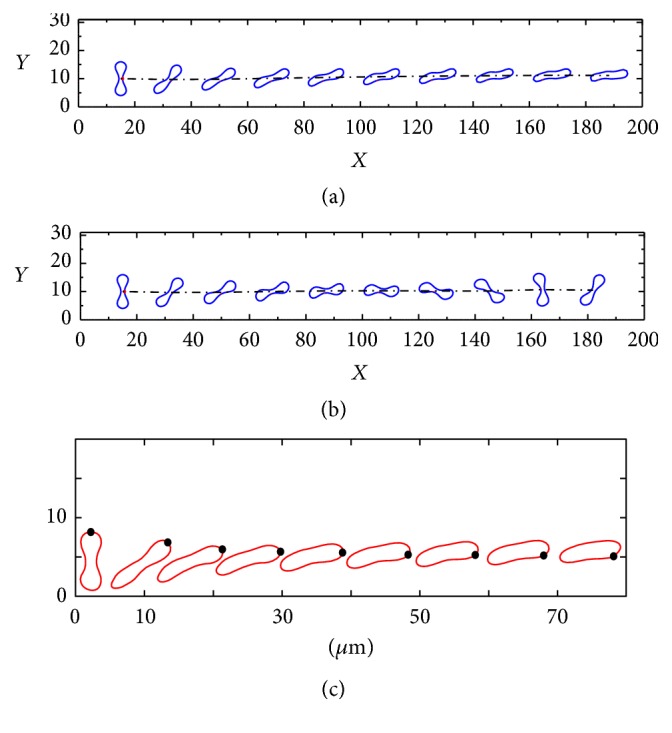
The time evolutions of the motions of RBC in Poiseuille flows, the initially vertical RBCs are positioned near the bottom lateral wall of the channel. (a) Healthy RBC, (b) sick RBC, and (c) numerical simulation by the FE-LBM [35].

**Figure 7:**
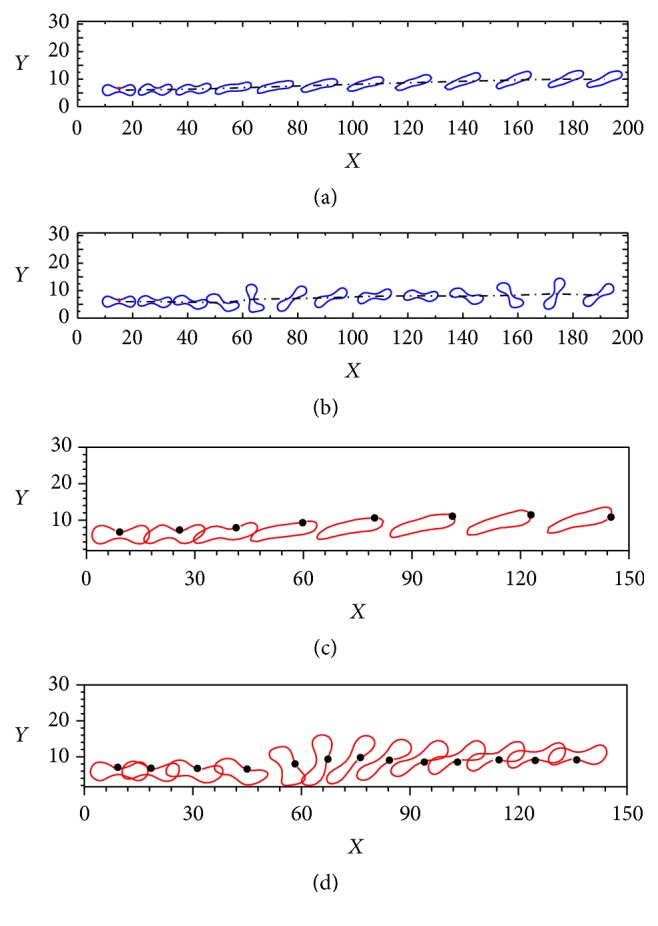
Rotational motions of healthy RBC ((a) and (c)) and sick RBC ((b) and (d)) in Poiseuille flows. The initially thwart-wise RBCs are positioned near the bottom lateral wall of the channel. ((a) and (b)) Current numerical simulation and ((c) and (d)) numerical results of [9].

**Figure 8:**
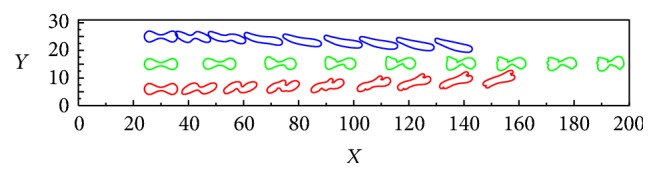
Rotational motions of three healthy RBCs asymmetrically positioned in the channel.

**Figure 9:**
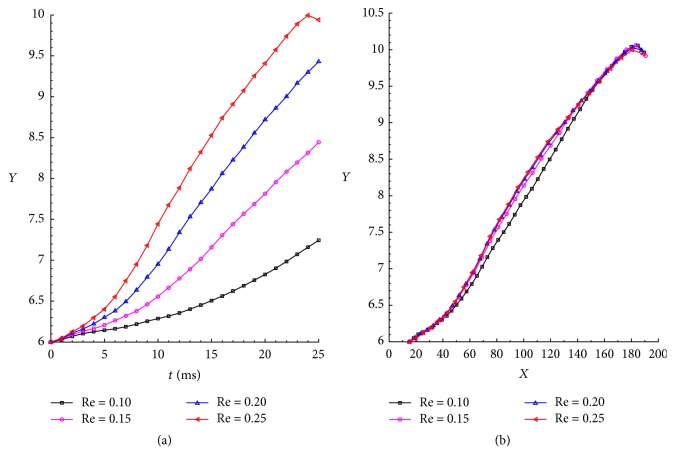
Variation of the RBC vertical movements with respect to the Re number of the flow: (a) the relationship of *t* and vertical distance and (b) the effect of Re on the barycentric coordinates.

**Figure 10:**
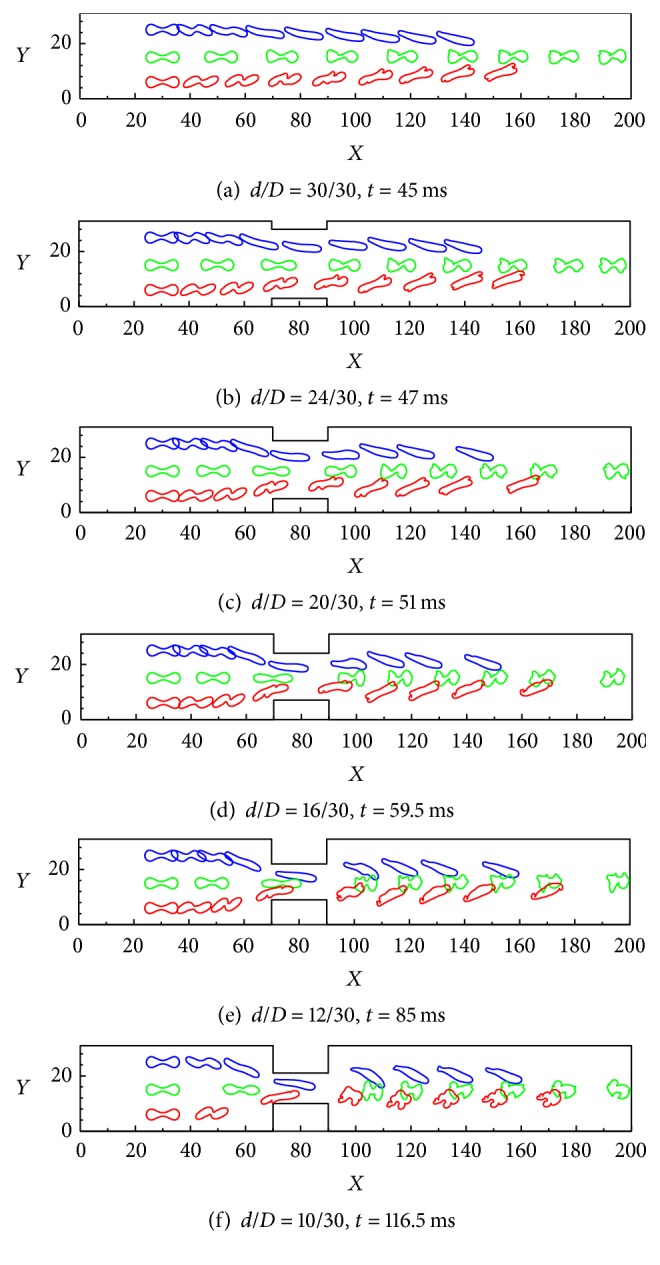
Transient deformations and motions of three healthy RBCs during Poiseuille flow through a constricted vessel: (a)* d/D* = 30/30 at* t* = 45 ms, (b)* d/D* = 24/30 at* t* = 47 ms, (c)* d*/*D *= 20/30 at* t* = 51 ms, (d)* d*/*D* = 16/30 at* t* = 59.5 ms, (e)* d*/*D* = 12/30 at* t* = 85 ms, and (f)* d*/*D* = 12/30 at* t* = 116.5 ms.

**Figure 11:**
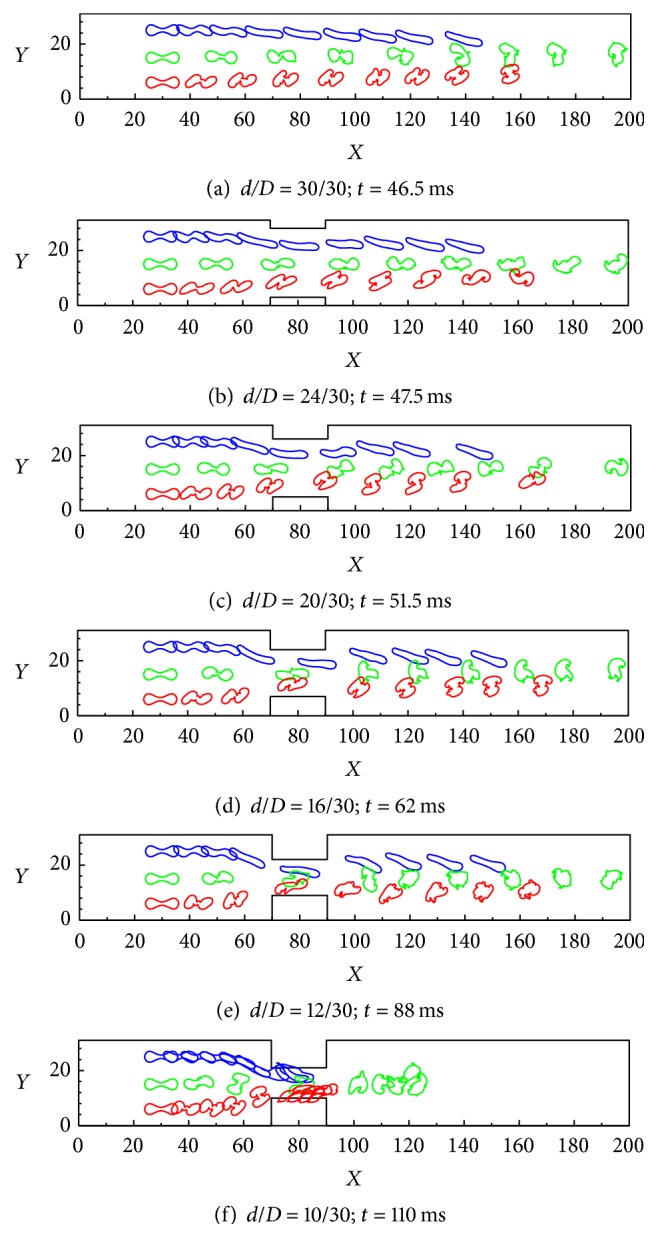
Transient deformations and motions of three sick RBCs during Poiseuille flow through a contracted vessel with Re = 0.10: (a)* d/D* = 30/30 at* t* = 46.5 ms, (b)* d/D* = 24/30 at* t* = 47.5 ms, (c)* d*/*D *= 20/30 at* t* = 51.5 ms, (d)* d*/*D* = 16/30 at* t* = 62 ms, (e)* d*/*D* = 12/30 at* t* = 88 ms, and (f)* d*/*D* = 12/30 at* t* = 110 ms.

**Figure 12:**
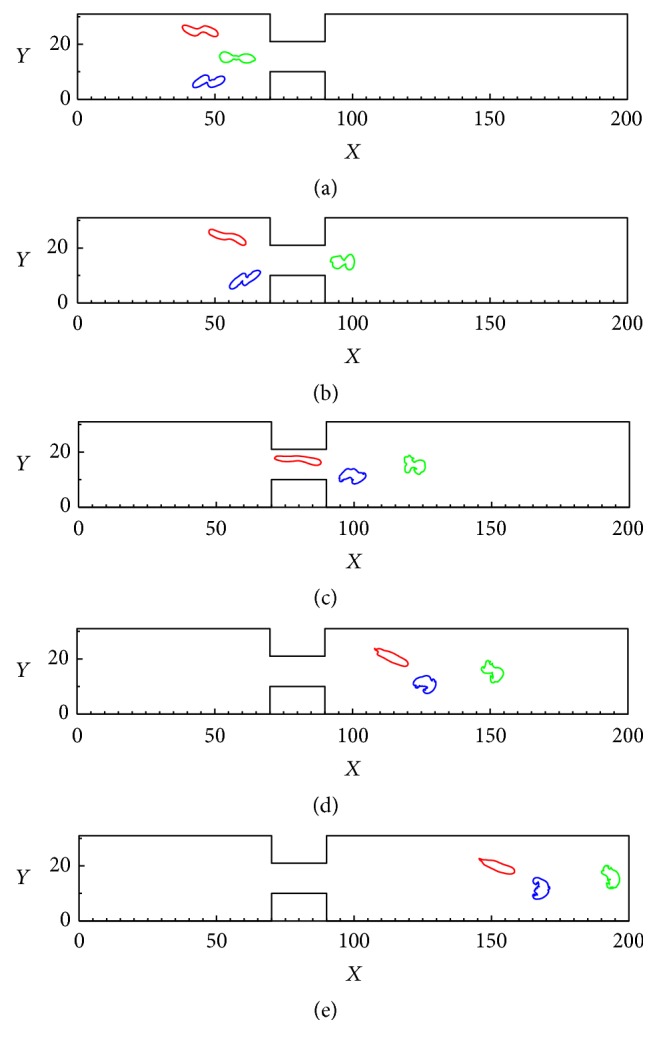
Deformations and motions of three sick RBCs in a constricted vessel (*d*/*D* = 10/30) with Re = 0.4.

To examine the effects of the degree of constriction on the motion and deformation of the RBCs, six cases with* d/D* values of 30/30, 24/30, 20/30, 16/30, 12/30, and 10/30 were, respectively, considered. The nondimensional ratios width-to-length (*W*/*L*), width-to-diameter (*W*/*R*), and length-to-diameter (*L*/*R*) were evaluated, where* W*,* L*, and *R* are the length, width, and radius of the RBCs, respectively (see Figure 4).

**Figure 13:**
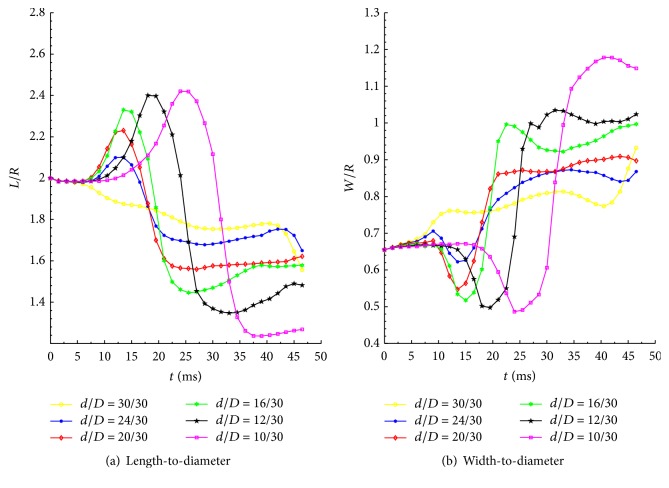
Variations of the (a) length-to-diameter and (b) width-to-diameter ratios of healthy RBCs during Poiseuille flow through microchannels with different degrees of constriction.

Figure 13 shows the variations of the ratios* L/R* and* W/R* for different degrees of constriction of the microchannel. It can be seen that the values of *L*/*R* and *W*/*R* were positively correlated with constriction ratio; the constriction area has a significant effect on the nondimensional parameters, and the RBCs undergo grater forced deformation compared to other areas of the microchannel to squeeze through the constriction. The peak position indicates that the RBCs have entered the constriction area. With the constriction ratio increasing, a longer time is required for the RBCs to squeeze through the constriction. When the RBCs pass through the constriction section, the RBCs regain a stable shape beyond the constriction area.

**Figure 14:**
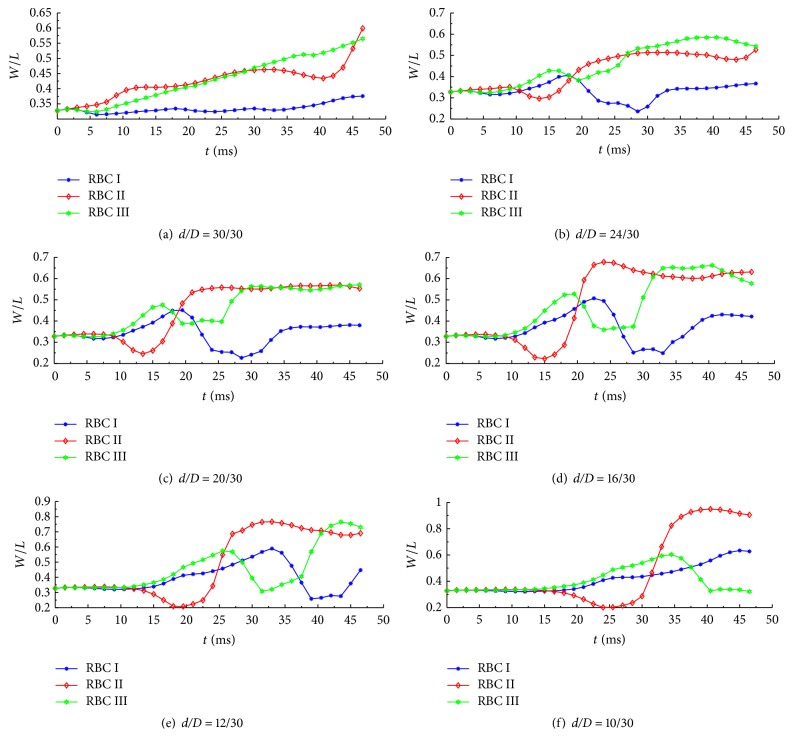
Variations of the width-to-length ratio for healthy RBCs during Poiseuille flow through microchannels with different degrees of constriction represented by* d*/*D* values of 30/30, 24/30, 20/30, 16/30, 12/30, and 10/30, respectively.

Figures 14(a)–14(f) show the variations of the ratio* W*/*L* of healthy RBCs for* d*/*D* values of 30/30, 24/30, 20/30, 16/30, 12/30, and 10/30, respectively. It can be seen from the figures that RBC II undergoes greater deformation and flows faster than RBCs I and III, and the peak position also indicates that the RBCs have entered the constriction area. This may be attributed to the shearing effect of the Poiseuille flow. Figures 14(b)–14(f) reveal that as the RBCs approach the constriction section, the nondimensional parameter* W*/*L* of RBCs has a substantial change until they leave the constriction area. RBC II (middle) moves quicker than RBC I and RBC III (lower), attributed to the effect of the Poiseuille flow. Another interesting phenomenon is that, with RBCs leaving constriction area, the W/L of RBC II has minor change, while the values for RBCs I and III abruptly decrease owing to the boundary effect.

**Figure 15:**
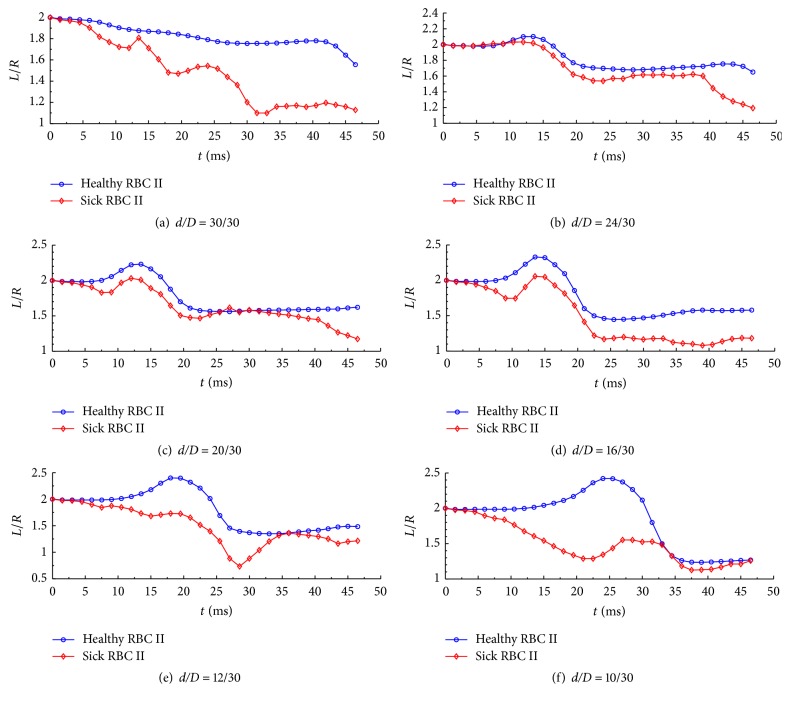
Variations of the length-to-radius ratio of the healthy and sick RBC II during Poiseuille flow through microchannels with different degrees of constriction represented by* d*/*D* values of 30/30, 24/30, 20/30, 16/30, 12/30, and 10/30, respectively.

The variations of* L/R* ratio for the healthy and sick RBCs II for different degrees of constriction are shown in Figure 15. It can be seen from the figures that the healthy RBC has a lower* L/R* ratio than the sick RBC. In addition, the variation of the* L*/*R* ratio of the healthy RBC is regular, whereas that of the sick RBC is irregular and oscillates.

## 4. Conclusions

The motions and deformations of three RBCs in a Poiseuille flow through a constricted microchannel were numerically investigated using the IB-LBM. The dynamics of the RBCs with respect to the degree of constriction of the microchannel, the Re number of the flow, and the elastic and bending moduli of the cells in the flow field were analyzed in detail. Following is a summary of the conclusions drawn from the observations.

Firstly, when the RBCs are located off the axis of symmetry of the microchannel, the shearing effect of the Poiseuille flow increases the forces acting on them, inducing their migration toward the centerline of the microchannel. Secondly, healthy RBCs exhibit higher deformability than sick RBCs during passage through a constriction area. In the process, the length-to-radius ratios of healthy RBCs vary regularly, whereas those of sick RBCs vary irregularly and oscillate. Thirdly, the width-to-radius and length-to-radius ratios of the RBCs increase with decreasing constriction ratio, with a longer time required for the RBCs to squeeze through a narrower constriction. However, the RBCs regain their stable shape beyond the constriction area. Furthermore, for sick RBCs in a microchannel with a constriction ratio as low as 1/3, adjustment of the flow parameters such as increasing the Re number to about 0.4 is required, given which they are able to pass and gradually migrate toward the centerline of the channel. This is particularly applicable to practical health conditions such as hemangioma and hypertension.

It is noteworthy that the present study only examined the effects of the degree of constriction of the channel and the Re number of the flow on the mechanical behavior of RBCs. Further study is required to examine the aggregation behavior of the cells and the elastic and viscoelastic effects of the channel. Additionally, for more accurate results, more sophisticated 3D models with more complex geometries such as bifurcation should be adopted for the simulations.
